# Main mechanisms and clinical implications of alterations in energy expenditure state among patients with pheochromocytoma and paraganglioma: A review

**DOI:** 10.1097/MD.0000000000037916

**Published:** 2024-04-26

**Authors:** Yuqi Yang, Tong Zhou, Xue Zhao, Yunjia Cai, Yao Xu, Xiaokun Gang, Guixia Wang

**Affiliations:** a Department of Endocrinology and Metabolism, The First Hospital of Jilin University, Changchun, China.

**Keywords:** energy expenditure, pheochromocytomas and paraganglioma (PPGL)

## Abstract

Pheochromocytoma and paraganglioma (PPGL) are rare neuroendocrine tumors with diverse clinical presentations. Alterations in energy expenditure state are commonly observed in patients with PPGL. However, the reported prevalence of hypermetabolism varies significantly and the underlying mechanisms and implications of this presentation have not been well elucidated. This review discusses and analyzes the factors that contribute to energy consumption. Elevated catecholamine levels in patients can significantly affect substance and energy metabolism. Additionally, changes in the activation of brown adipose tissue (BAT), inflammation, and the inherent energy demands of the tumor can contribute to increased resting energy expenditure (REE) and other energy metabolism indicators. The PPGL biomarker, chromogranin A (CgA), and its fragments also influence energy metabolism. Chronic hypermetabolic states may be detrimental to these patients, with surgical tumor removal remaining the primary therapeutic intervention. The high energy expenditure of PPGL has not received the attention it deserves, and an accurate assessment of energy metabolism is the cornerstone for an adequate understanding and treatment of the disease.

## 1. Introduction

Pheochromocytoma and paraganglioma (PCC/PGL, collectively PPGL) are relatively rare endocrine tumors originating from the adrenal medulla and extra-adrenal sympathetic chain, respectively. They are characterized by excessive secretion of catecholamines including norepinephrine, epinephrine, and dopamine, leading to clinical symptoms including high blood pressure; dizziness; palpitations; excessive sweating; and potentially severe complications affecting the heart, brain, and kidneys. Patients may also present atypical symptoms including fatigue, nausea, vomiting, chest and abdominal pain, blurry vision, constipation, fever, and weight loss.^[1]^

Resting energy expenditure (REE), a substitute for basal metabolic rate (BMR), is the largest and most frequently monitored component of total energy expenditure. Hypermetabolism is clinically defined as an estimated resting energy expenditure to a predicted resting energy expenditure ratio of ≥ 110%.^[2]^ In this state, the caloric, nutrient, and oxygen requirements increase to accommodate the heightened energy demands and nutrient turnover of cells and tissues. Various factors can cause hypermetabolism, including sepsis, burns, and trauma,^[3]^ as well as hyperthyroidism and some cancers.

Patients with PPGL have an elevated BMR,^[4–6]^ which is occasionally misdiagnosed as concomitant hyperthyroidism with PCC. However, relatively few studies have directly measured energy consumption indicators in patients with PPGL. Additionally, weight loss, a typical manifestation of energy expenditure exceeding intake, is also common among patients with PPGL and is sometimes a primary reason for their medical visits.^[7]^ Discrepancies in energy intake, whether deficient or excessive, can aggravate these imbalances, leading to altered body composition and negative effects on disease prognosis.

Generally, compared with classical cardiovascular manifestations, the signs and symptoms related to hypermetabolism in patients with PPGL are milder and less likely to attract the attention of patients and medical professionals. Therefore, this review discusses and analyzes the potential mechanisms and evidence of hypermetabolism in PPGL and the role of hypermetabolism in disease progression and clinical decision-making.

## 2. Methods

In this narrative review, we searched the PubMed database to identify relevant literature using search terms including “pheochromocytoma and paraganglioma,” “catecholamines,” “energy expenditure,” “substance metabolism,” “weight changes,” “brown adipose tissue,” “inflammation,” “tumor cachexia,” and “ chromogranin A (CgA)” Only articles relevant to the factors and effects of high energy expenditure on PPGL discussed in this study were included.

## 3. Abnormal energy metabolism in patients with PPGL

Hypermetabolic states are common among patients with PPGL. The prevalence of hypermetabolic states according to REE is approximately 49% to 70%.^[8–10]^ Previous studies used BMR to describe increased metabolism, with values in patients with PCC varying widely from −9% to +80% of the predicted value.^[11]^ REE has shown increments ranging from +10.4% to +16%,^[8–10]^ with significant reductions after adrenalectomy, and good consistency across studies. Earlier studies using closed-circuit apparatuses to calculate BMR tended to overestimate BMR^[12]^; in contrast, REE values computed using computerized, open-circuit, and indirect calorimetry were close to the true BMR levels. Recent studies have reported mean REE values without providing specific REE measurements and predictions for each patient with PPGL; however, some patients have low or normal metabolic statuses. Regarding changes in body weight, studies focusing primarily on weight outcomes are limited, and varying degrees of weight loss have been reported. In individual case reports, patient weight loss was significant (>10 kg), mostly in elderly women.^[7,13–16]^ Among 79 patients with PCC, four cases (5.1%) demonstrated a preoperative weight loss of > 10%.^[17]^ Other studies reported weight loss rates of 10.5% to 6% (including children) but did not provide specific descriptions of the extent of weight loss.^[18–20]^ Preoperative BMI and weight are lower in patients with PCC and hypertension, as well as in those with PPGL.^[21,22]^ Preoperative weight and BMI are significantly lower in patients with PCC than in patients with adrenal aldosterone-producing adenomas.^[23]^ Moreover, patients with PCC have lower BMI than patients with nonfunctional adrenal adenoma and primary hypertension.^[24,25]^ In contrast, patients with PCC showed a higher BMI than those with primary hypertension and healthy control groups.^[10,26]^ The proportion of women with PPGL was relatively higher in some studies. A study investigating the clinical differences between benign and malignant PCC in 58 patients revealed significantly higher rates of reported weight (88% vs 43%) and energy level (89% vs 64%) changes in women than among men.^[27]^ Surgical removal of endocrine tumors corrected the abnormal energy expenditure state, leading to varying degrees of weight gain and increased BMI. The timing of weight recovery is influenced by the monitoring frequency, although patient weight presumably starts to increase immediately after tumor removal^[28]^ (Table 1). Hence, the hypermetabolic state in patients with PPGL is not an individual-specific trait but rather a generally prevalent phenomenon.

**Table 1:** Preoperative and postoperative change in weight of patients with pheochromocytoma and paraganglioma.

Author	PPGL group (n = 1253)		Preoperative weight (kg) or BMI (kg/m^2^)		Postoperative weight (kg) or BMI (kg/m^2^)		Control group (n = 1633)	Weight (kg) BMI (kg/m^2^)	Follow-up time	Factors of weight/BMI change
2010 Elenkova A^[26]^	26		23.9 ± 3.13				30 (EH) 31 (HS)	21.9 ± 1.7 22.33 ± 1.97	6–18 mo	
	10	10^F^	23.8 ± 2.2	26.22 ± 2.3^F^	26.4 ± 2.2*	29.32 ± 4.5^F^*				
2015 Okamura T^[24]^	42		22.2 ± 2.9		23.4 ± 3.4**		23	23.2 ± 2.4		
2016 Lu Y^[21]^	65		61.4 ± 13.7 22.5 ± 3.8				95	65.6 ± 5.4* 24.8 ± 2.5*		
2017 Majtan B^[25]^	50		26.8 ± 5.2 78 ± 15		82 ± 15***		50	27.7 ± 4.9	5.0 ± 0.9 yr	
2017 Spyroglou A^[23]^	43		68.0 ± 15.0 23.7 ± 4.3		71.0 ± 15.8*** 24.7 ± 4.6***		176	84.5 ± 17.6*** 28.6 ± 5.0***	1 yr	U-NMN
2019 An Y^[22]^	210		25.1 ± 0.4 24.9		25.7****		1158	28.1 ± 0.2****	13 mo	U-CA; MN
2021 Krumeich L.N^[28]^	360		27.4 82.2		83.2				54.2 mo	NMN
2022 Petrák O^[10]^	108		26.3 ± 5.5 77 ± 20				70	25.7 ± 4.2 75 ± 15		
2022 Araujo-Castro M^[29]^	229				Δ + 3.13kg***				12 mo	

BMI = body mass index, ES = Essential hypertension, F = Female, HS = healthy subjects, PCC = Pheochromocytoma, PGL = Paraganglioma, PPGL = pheochromocytoma and paraganglioma, U-CA = urine catecholamine, U-MN = urine normetanephrine, U-NMN = urine normetanephrine, Δ = change in weight.

* *P* < .05.

** *P* < .01.

*** *P* < .001.

*** *P < .0001.

## 4. Mechanisms of hypermetabolism in patients with PPGL

**Figure 1.**
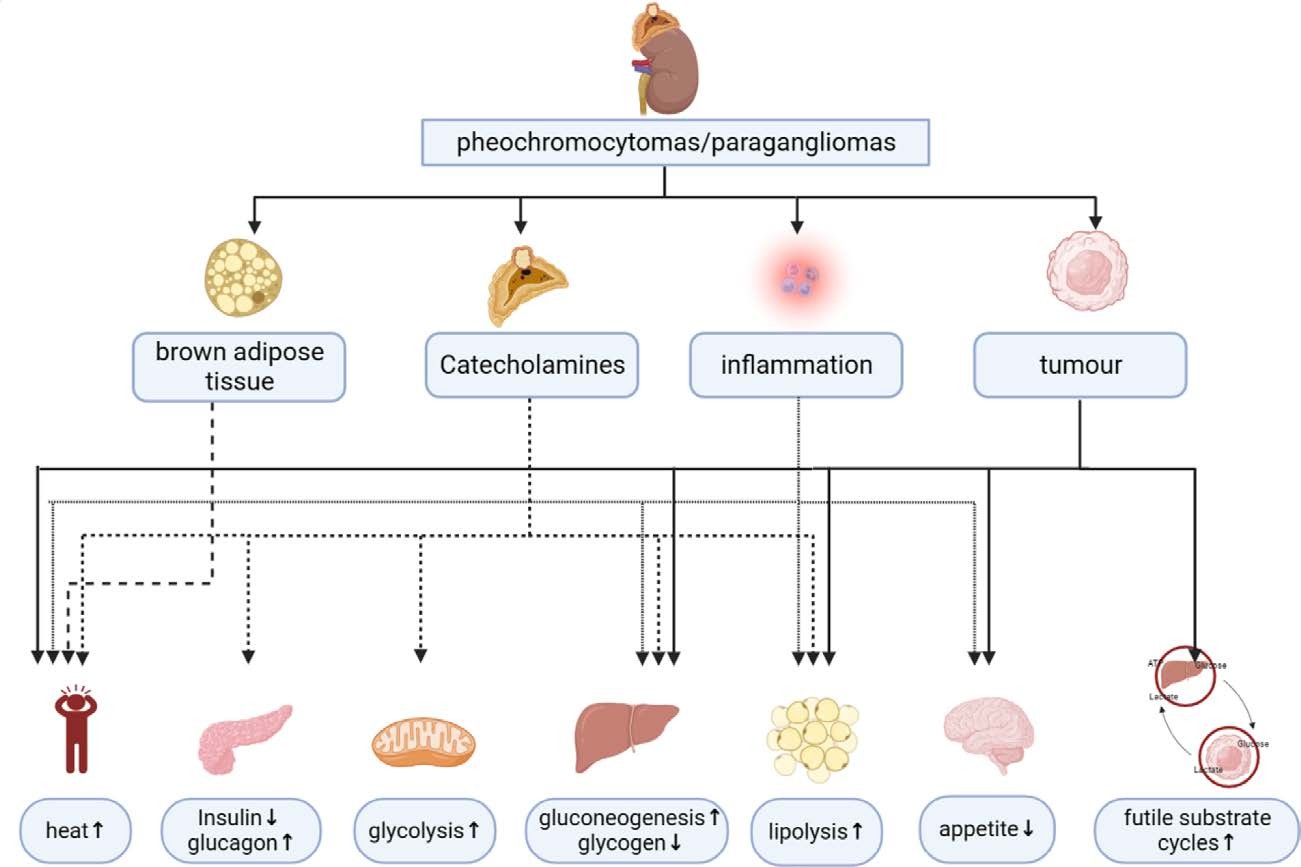
Impacts of PPGL on energy metabolism. PPGL = pheochromocytomas and paragangliomas.

The reasons for increased metabolism in patients with PPGL are not singular, but the detailed associated mechanisms remain unclear. First, the excessive catecholamine secretion in patients with PPGL may be an important mechanism leading to increased metabolic activity in organs and tissues. Second, patients with PPGL also exhibit a higher rate of brown adipose tissue (BAT) activation than normal individuals. BAT increases energy expenditure in the form of characteristic thermogenesis. Furthermore, inflammation stimulates energy expenditure. In addition, the expression of the classic biomarkers of PPGL, CgA and its derivatives, is associated with substance and energy metabolism. Finally, considering the malignant potential of PPGL, the impact of tumors themselves on body metabolism also requires consideration (Fig. 1).

### 4.1. Catecholamines enhance substance metabolism in patients with PPGL

Catecholamines significantly impact substance and energy metabolism. The heightened metabolic state induced by catecholamines is positively correlated with increased oxidation and consumption of carbohydrates, proteins, and fat.^[30,31]^ Under physiological conditions, catecholamines induce glycolysis, promote gluconeogenesis and glycogenolysis, and inhibit insulin-mediated glycogen synthesis. Catecholamines also have thermogenic properties, which are associated with increased glucose oxidation; relatively high-calorie production from fatty acid oxidation; and modulation of the secretion of other synthesis/degrading hormones such as insulin, cortisol, and glucagon.^[32]^ The metabolic effects of catecholamines are mainly mediated by the activation of adenylyl cyclase through β-receptor stimulation, leading to cAMP accumulation.^[33]^ Catecholamines exacerbate metabolic hyperactivity by promoting hyperglycemia and hyperlactatemia in stress states such as sepsis and shock.^[34]^ In contrast, in obesity, the adipose tissue is resistant to catecholamines and shows reduced energy expenditure, while increased adipose catecholamines prevent obesity in mice.^[35]^

The excessive secretion of catecholamines in PPGL is undeniable. With the removal of PPGL tumors and the normalization of catecholamine levels, the patient’s hypermetabolic state is corrected. No definitely correlations among calorimetry parameters and hormones were detected^[8]^, but a significant correlation between BMI changes and urinary normetanephrines was observed.^[23]^

Patients with PPGL often show impaired glucose tolerance. The incidence of diabetes among patients with PPGL ranges from 21% to 37%,^[36]^ with a higher prevalence of impaired glucose tolerance (approximately 49.5%).^[37]^ Diabetic ketoacidosis and hyperosmolar hyperglycemic coma can also occur.^[29,38]^ The main reasons for impaired glucose tolerance in PPGL are impaired insulin secretion and increased insulin resistance.^[39]^ Moreover, different catecholamine secretion phenotypes in PPGL have varying effects on impaired glucose tolerance. Abe et al suggested that excessive adrenaline primarily impairs insulin secretion, whereas noradrenaline primarily increases insulin resistance. This might be due to adrenaline’s high affinity for α2 receptors to stimulate pancreatic β-cells, leading to reduced insulin secretion, and noradrenaline’s high affinity for α1 and β3 receptors to stimulate pancreatic α-cells and adipocytes, leading to increased glucagon and free fatty acids (FFAs) levels, thus increasing hepatic glycogen synthesis and breakdown.^[40]^ Recent studies indicate that the risk factors for preoperative glucose abnormalities include older age,^[37,41–43]^ hypertension comorbidity,^[42–44]^ symptomatic tumors,^[42,45,46]^ longer disease duration,^[37]^ larger tumor size,^[42]^ 24-hour urinary norepinephrine^[37,43]^ or epinephrine levels,^[43]^ and sporadic PPGL^[44]^ (Table 2). Additionally, following tumor removal, many patients with PPGL experience recovery or partial relief of impaired glucose tolerance.

**Table 2:** Preoperative and postoperative change in glycemic status of patients with pheochromocytoma and paraganglioma.

Author	PPGL (n = 682)	Prevalence of DM, %	Factors of DM progress	Remission Rate of DM, %	Follow-up time
2017 Beninato T^[46]^	153	23.4	Larger tumors; symptomatic tumors;	78.6	52.1 mo
2019 Liu Z^[41]^	185	36.2	Higher age; a longer course of PCC	61.7	45.2 mo
2020 Elenkova A^[37]^	62	30.4	Higher age; higher U-MN and NMN	60	5 yr
2020 Khatiwada S^[42]^	37	48	Higher age; higher number of anti-hypertensive agent; secretory tumors	77	4 mo
2021 Sara Derrou^[45]^	23	26	Lager tumors; symptomatic tumors	33.33	1 yr
2022 Araujo-Castro M^[44]^	229	23.6	Sporadic PPGL; hypertension	51.9	56.8 mo
2023 Zhao L^[43]^	163	35.6	Higer age; hypertension; higher U-E	63	20.55 mo

DM = Diabetes Mellitus, PCC = pheochromocytoma, PPGL = pheochromocytoma and paraganglioma, U-E = urine epinephrine, U-MN = urine normetanephrine, U-NMN = urine normetanephrine.

Lipids, another crucial aspect of substance metabolism and energy sources, exhibit changes in patients with PPGL that are not as well defined as elevated blood glucose levels. According to the principle of catecholamine-promoting lipolysis, patients with PPGL should have reduced fat and blood lipid levels. Patients with PPGL show significant increases in subcutaneous fat tissue area and visceral fat tissue area (VFA) after surgery.^[24]^ Moreover, computed tomography after adrenal resection in patients with PCC had 14.5% and 15.8% higher distributions of VFA and subcutaneous fat tissue area, respectively.^[47]^ Increased FFA levels have also been reported in patients with PPGL, predominantly in case series reports.^[48]^ Conversely, another study reported a 46.3% incidence of hyperlipidemia in patients with PCC, including hypertriglyceridemia (18.5%), hypercholesterolemia (24.1%), low-density lipoproteinemia (16.6%), and low-high-density lipoprotein cholesterolemia (16.6%).^[49]^ Although another study did not observe a significant difference in total cholesterol and triglyceride levels between the PPGL and control groups, statin use was significantly higher in the control group.^[10]^ The blood lipid profiles of patients with PCC before and after surgery demonstrated no significant changes in various lipid indicators with normalization of catecholamine levels.^[26,50]^ Okamura et al^[24]^ demonstrated that postoperative triglycerides and low-density lipoprotein cholesterol levels showed no significant changes; however, unexpectedly, the preoperative HDL level was positively correlated with urinary noradrenaline levels and decreased significantly after surgery. However, the direct effect of catecholamines on HDL involves complex mechanisms. Okamura’s findings align with those by Ward and Dai,^[51,52]^ possibly indicating a “protective” effect of catecholamines on hyperlipidemia, wherein catecholamines promote fat breakdown, leading to the excretion of cholesterol from fat cells to the extracellular membrane, effectively resulting in “lipid reduction” with HDL^[53]^ (Table 3).

**Table 3:** Preoperative and postoperative change in lipid levels of patients with pheochromocytoma and paraganglioma.

Author	PPGL (n = 234)		Preoperative value (mmol/L)	Postoperative value (mmol/L)	Control (n = 103)	Lipid value	Follow-up time
2008 Bernini G^[50]^	10	TC	5.35 ± 0.24 mmol/L	5.10 ± 0.70 mmol/L	10	5.30 ± 0.60 mmol/L	31.5 ± 2.2 mo
		TG	1.55 ± 0.28 mmol/L	1.42 ± 0.19 mmol/L		1.46 ± 0.30 mmol/L	
		LDL	3.06 ± 0.22 mmol/L	3.29 ± 0.15 mmol/L		3.30 ± 0.18 mmol/L	
		HDL	1.62 ± 0.11 mmol/L	1.53 ± 0.60 mmol/L		1.52 ± 0.40 mmol/L	
2010 Elenkova A^[26]^	10 10 ^F^	TC	4.63 ± 0.66 mmol/L 5.29 ± 0.92 mmol/L ^F^	4.39 ± 1.07 mmol/L 4.87 ± 0.95 mmol/L ^F^			6–18 mo
		TG	1.10 ± 0.37 mmol/L 1.68 ± 1.02 mmol/L ^F^	0.93 ± 0.45 mmol/L 1.63 ± 0.61 mmol/L ^F^			
		HDL	1.25 ± 0.14 mmol/L 1.6 ± 0.7 mmol/L ^F^	1.29 ± 0.36 mmol/L 1.45 ± 0.77 mmol/L ^F^			
2015 Okamura T^[24]^	42	TG	108 ± 87 mg/dL	120 ± 80 mg/dL	23	106 ± 42 mg/dL	612 mo
		LDL	115 ± 28 mg/dL	105 ± 22mg/dL		103 ± 24 mg/dL	
		HDL	60 ± 18 mg/dL	55 ± 16 mg/dL**		57 ± 13 mg/dL	
2020 Good M.L^[49]^	54	TC	176.4 ± 34.78 mg/dL	166.3 ± 35.53mg/dL**			3.1 mo
		TG	117.2 ± 67.8 mg/dL	113.2 ± 84.2 mg/dL			
		LDL	97.0 ± 31.5 mg/dL	97.0 ± 31.5 mg/dL			
		HDL	56.7 ± 16.6 mg/dL	53.2 ± 14.7 mg/dL*			
2022 Petrak O^[10]^	108	TC	4.7 ± 1.1 mmol/L		70	4.8 ± 0.9 mmol/L	
		TG	1.3 ± 0.8 mmol/L			1.6 ± 1.4 mmol/L	

F = female, HDL = high-density lipoprotein cholesterol, LDL = low-density lipoprotein cholesterol, PPGL = pheochromocytoma and paraganglioma, TC = total cholesterol, TG = triglycerides.

* *P* < .05.

** *P* < .01.

Regarding substrate utilization in the hypermetabolic state of PPGL, heat index calculations and 24-hour urinary nitrogen showed that the noradrenergic phenotype primarily metabolized carbohydrates, whereas the adrenergic phenotype primarily metabolized lipids. However, the protein metabolism and incidence of hypermetabolic states did not differ significantly between the two phenotypes. The authors speculated that the molecular mechanism of adrenergic receptors and genetic mutations in PPGL may explain this phenomenon.^[10]^ However, the authors did not further investigate genetic mutations in the tumor; thus, this hypothesis remains unverified. Future research should focus on the specific mechanisms of metabolic hyperactivity in tumors with different secretion phenotypes. Under the influence of the disease, the reactivity of adrenergic receptors changes,^[54,55]^ leading to an unpredictable response of metabolic processes to catecholamine stimulation.

### 4.2. BAT contributions to hypermetabolism

BAT activation can also influence the metabolic state in PPGL. BAT is a specialized type of fat that increases energy expenditure through heat production. Brown adipocytes are characterized by numerous mitochondria and multiple small lipid droplets. BAT is rich in blood vessels that ensure efficient heat dissipation during thermogenesis.^[56]^ When sympathetic nerves release catecholamine substances, they activate β3-adrenergic receptors on brown adipocytes, initiating lipolysis of stored triglycerides within the cells. This process releases FFAs as substrates, which activate mitochondrial oxidative respiration. Simultaneously, the activation of uncoupling protein-1 (UCP-1) leads to the uncoupling of electron transport and ATP production, releasing a significant amount of energy in the form of heat.^[57]^ Apart from cold stimulation, other factors such as adrenergic stimulation (norepinephrine), thyroid hormone stimulation, bile acids, fibroblast growth factor 21 (FGF21), and irisin activate BAT and induce thermogenesis.^[58]^ A preliminary study showed that the administration of high-dose β3 adrenergic receptor agonists to healthy men, under conditions of fully activated BAT, resulted in an average increase of 203 ± 40 kcal/d in REE,^[59]^ indicating the potential of BAT activation to increase energy expenditure.

In patients with PPGL, individual case reports and population-based studies confirmed BAT activation through imaging, pathology, and genetic analyses. The activated sites of BAT include not only typical regions such as the neck, supraclavicular, mediastinal, paravertebral, renal and adrenal perirenal, and pericardial areas but also atypical areas such as the omentum and mesentery.^[60–66]^ In the general population, the activation rate of BAT is approximately 3.1% to 7.5%.^[67]^ ^18^F-fluorodeoxyglucose (FDG)-positron emission tomography/computed tomography (PET/CT) examinations showed a BAT activation rate of approximately 27.4% in patients with PPGL, compared to approximately 6.1% in patients without PPGL.^[68]^ A study in the US reported similar results, with positivity rates of 22% and 9.5% for PCC and non-PCC, respectively.^[69]^

Previous studies reported higher plasma catecholamine or its metabolite levels in the BAT-activated group of patients with PPGL than in the control group.^[65,69,70]^ In contrast, another study reported no difference between BAT-activated and non-activated groups, although the study included patients with non-secretory PPGL without BAT activation.^[63]^ In a study investigating BAT gene expression in patients with PPGL, the mRNA levels of related genes were positively correlated with catecholamine metabolites and decreased with age and BMI, consistent with the general population.^[61]^ These studies suggest that the high concentration of circulating catecholamines in patients with PPGL leads to excessive sympathetic nervous system activation and subsequently triggers BAT activation, rather than the disease itself. Additionally, follow-up imaging after surgical removal of PCC revealed regression of BAT activation, implying that surgical removal of the source of catecholamine secretion might attenuate sympathetic nervous system stimulation and the disappearance of BAT activation.^[66]^ However, as BAT activation also occurs in individuals with normal circulating catecholamine levels, other factors may also induce BAT activation in patients with PPGL, such as elevated FGF21 levels.^[9,71]^

BAT activation as a mechanism of metabolic hyperactivity in patients remains controversial. In one case report, ^18^F-FDG-PET/CT showed that a patient with PCC had a REE more than twice the predicted value before surgery, revealing significant infiltration of visceral fat with BAT. After surgery, the energy expenditure returned to normal along with increased weight.^[72]^ However, in a study of 25 patients with PPGL and 14 control patients, Klimova et al reported significant increases in the mRNA expression levels of CCAAT enhancer binding protein beta (CEBPB), iodothyronine deiodinase 2 (DIO2), peroxisome proliferator-activated receptor gamma coactivator 1-alpha (PPARGC1A), and UCP-1 in VFA, which are associated with brown and beige fat, along with their relationship to catecholamines and clinical thermogenesis. Although > 60% of the patients were hypermetabolic, no direct relationship was found between UCP1-related gene expression and the baseline energy metabolism parameters determined by indirect calorimetry.^[61]^ However, gene expression does not equate to protein expression, and further investigation is needed to confirm whether BAT activation truly exists in patients with hypermetabolic PPGL or to conduct further subgroup analysis to ascertain whether patients with hypermetabolic PPGL have higher levels of BAT activation-related gene expression. Another study by the same researchers showed that successful surgery reduced FGF21 levels but observed no correlation between FGF21 and REE.^[9]^ However, serum FGF21 levels may not represent its production and activity in local adipose tissue. Exploring precise methods for measuring active FGF21 as well as tissue biopsies may provide more direct evidence. Additionally, excess catecholamines can lead to adrenergic receptor desensitization, eliminating the action of FGF21. Insulin resistance can inhibit glucose uptake by BAT, and the precise assessment of BAT activation levels in PPGL patients with insulin resistance is also limited in current research. The available literature mainly consists of case reports, with a limited number of studies directly measuring energy expenditure indicators, resulting in a limited level of evidence. Moreover, BAT activity is influenced by various intrinsic and extrinsic factors, such as environmental temperature, age, sex, medications, and other comorbidities. Therefore, BAT activation may be one of the mechanisms contributing to the increased energy expenditure in patients with PPGL.

### 4.3. Regulation of energy metabolism by inflammation among patients with PPGL

Chronic low-grade inflammation can also increase energy expenditure in patients with PPGL, resulting in metabolic hyperactivity. Research on the influence of inflammation on energy expenditure has primarily focused on obesity, burns, and cancer cachexia. In obesity, the impact of inflammation on metabolism has traditionally been considered negative because the adipose tissue responds to overnutrition by initiating an immune response that is often accompanied by insulin resistance. However, inflammation is also a critical factor in regulating energy balance. Inflammation indirectly promotes energy expenditure by inducing leptin and glucagon-like peptide 1 (GLP-1) expression, while promoting white adipose tissue (WAT) browning and lipolysis. In cachexia, tumors directly produce or trigger the production of inflammatory factors, which inhibit appetite, consume muscle protein, promote lipolysis, activate BAT, or induce WAT browning, ultimately leading to energy expenditure exceeding energy intake.^[73]^ In burns, stress mediates an increase in the levels of systemic inflammatory factors, which, combined with catecholamines and glucocorticoids, drive a hypermetabolic state by increasing the turnover of glucose, fat, and protein throughout the body.^[74]^ In 2000, Suttmann et al investigated the relationship between body composition, REE, and inflammatory factors such as interleukin 6 (IL-6) and tumor necrosis factor (TNF) in 12 patients with HIV-associated infections. The authors reported a 34% increase in REE compared with the expected values, and the deviation of REE measurements from the predicted values correlated with the concentrations of TNF and IL-6. Moreover, TNF is significantly correlated with the excretion of adrenal glands and noradrenaline in urine^[75]^

Patients with PPGL show elevated circulating levels of inflammatory markers. PPGL produces catecholamines and other neuropeptides and hormones, such as IL-6. Several cases of PPGL in patients with elevated interleukin-6 (IL-6) levels have been reported; these patients often exhibit symptoms including fever of unknown origin, weight loss, anemia, thrombocytosis, and systemic inflammatory response syndrome. The clinical manifestations and laboratory results of these patients returned to normal after the successful removal of the pheochromocytoma or paraganglioma.^[15,76–79]^ Several cohort studies in patients with PCC have reported significantly higher white blood cell and platelet counts as well as elevated levels of acute-phase reactants such as C-reactive protein (CRP), compared to the control group. After adrenalectomy, white blood cell count, TNF-α, IL-6, and interleukin-8 (IL-8) levels decreased significantly.^[8,80]^

As mentioned above, PPGL itself can produce inflammatory cytokines such as IL-6 or TNF-α. Catecholamines can also induce inflammation, induce leukocytosis, increase platelet counts, and activate platelets.^[81,82]^ Stress hormones can induce IL-6, IL-8, TNF-α, and CRP production.^[83]^ In a recent comparative study of patients with PCC and primary hypertension, catecholamines induced persistent pro-inflammatory changes in monocytes, both in vitro and in vivo.^[84]^ Moreover, the presence of tumors may lead to immune activation and increased inflammation. Chronic catecholamine excess in patients is associated with significantly increased levels of inflammatory markers (white blood cells, platelets, and CRP), which are significantly higher than those in patients with primary hypertension and aldosteronism. Even after catecholamine normalization post-surgery, the levels of inflammatory markers did not completely normalize but remained higher than those in the control group, suggesting delayed recovery of pro-inflammatory factors, and catecholamines, in PCC.^[80]^ Bosanska et al^[85]^ also observed decreased CRP levels after PCC surgery but did not explore the relationship between CRP and catecholamines and their metabolites.

Regarding the relationship between inflammation and energy metabolism in patients with PPGL, a retrospective analysis of 29 patients with pheochromocytoma (7 with paroxysmal symptoms, 22 without) revealed significantly higher IL-6 and CRP expression levels in those with paroxysmal symptoms.^[86]^ However, a study on patients with PCC did not find significant correlations between indirect calorimetry parameters like REE and serum cytokines or inflammatory markers (white blood cell count, TNF-α, IL-6, IL-8).^[8]^ Thus, the roles and mechanisms of inflammatory factors in the energy metabolism of patients with PPGL require clarification.

### 4.4. Effects of chromaffin tumors within or outside of the adrenal gland on metabolism

The hypermetabolic state in PPGL may indicate increased tumor activity. Various types of cancer tend to show normal or increased REE.^[87–89]^ One reason for tumor-induced abnormal REE is the ineffective circulation of substrates. The high metabolic state of the tumor leads to intratumoral hypoxia, resulting in increased glycolysis and lactate production. Excess lactate is converted back to glucose in the liver, leading to the net consumption of ATP. Another contributing factor is the uncoupling of oxidative phosphorylation, leading to inefficient energy utilization and increased heat dissipation. UCP expression is significantly increased in cachexia, as well as in the uncoupling of sarcoplasmic reticulum Ca^2+^-ATPase and membrane Na^+^-K^+^-ATPase. Tumor-induced activation of the immune system leads to increased levels of inflammatory mediators such as IL-6, which contribute to changes in the hypothalamic-pituitary axis, ineffective substrate circulation, and uncoupling of oxidative phosphorylation, resulting in increased REE and decreased appetite. BAT activation or WAT browning has also been observed in patients with cachexia, possibly due to the action of tumor-derived parathyroid-related proteins and inflammation. Liver metastasis from the tumor can also lead to abnormal REE. Increased liver metastatic mass is accompanied by increased REE, usually in the late stages of tumor development.^[90–92]^

Approximately 10% of PCC and 25% of extra-adrenal PGL in PPGL are metastatic, with the highest metastatic potential seen in hereditary succinate dehydrogenase subunit B (SDHB)-related PPGL, where the proportion can reach up to 50%.^[93]^ In 2017, the World Health Organization eliminated the term “benign pheochromocytoma,” asserting that all pheochromocytomas have malignant potential.^[94]^ Therefore, cachexia should be considered among factors contributing to the hypermetabolic state in patients with PPGL.

However, few studies have compared the differences in energy metabolism between patients with metastatic and non-metastatic PPGL. Perhaps, with a thorough understanding of the malignant features of PPGL, the influence of tumors on PPGL can be clearly defined and quantified. Further research to fill these gaps is eagerly anticipated.

### 4.5. Chromogranin A and its fragment pancreastatin may influence energy metabolism

CgA, a biomarker for neuroendocrine tumors,^[95]^ is an acidic glycoprotein that generates various biologically active peptides upon intragranular or extragranular hydrolysis, including the peptides CgA1-76 (vasostatin-I), CgA1-113 (vasostatin-II), CgA79-113 (vasoactive inhibitory peptide, VIP), CgA250-301 (pancreastatin), CgA352-372 (catestatin), CgA411-436 (serpinin), and more.^[96]^ Studies on the substance and metabolism of CgA and its components mainly focus on pancreatin (PST). PST was initially thought to be a novel insulin secretion inhibitory peptide^[97]^ and was later confirmed to be generated by the hydrolysis of CgA.^[98]^ In rodents, PST significantly affects substance and energy metabolism, particularly glucose and lipid metabolism, resulting in insulin resistance. PST inhibits insulin secretion from β cells,^[99]^ mediates insulin resistance by reducing glucose uptake and utilization in adipocytes,^[100]^ inhibits hepatic glycogen synthesis,^[101]^ and stimulates glycogen breakdown in the liver.^[102]^ Lipid metabolism increases adipocyte lipolysis.^[100]^ The effect of protein metabolism is less pronounced and stimulates protein synthesis.^[103]^ PST reduces leptin expression and enhances UCP-2 expression^[104]^ but has no effect or inhibits UCP-1 expression^[104,105]^ or peroxisome proliferator-activated receptor gamma (PPAR-γ).^[104]^ In humans, elevated CgA and PST levels have been observed in metabolic diseases such as hypertension^[106]^ and type 2 diabetes.^[107]^ The intravenous injection of PST into the forearm reduced glucose uptake and promoted fatty acid release in the human body without affecting amino acids.^[108]^ CgA and its derivatives have garnered significant attention as potential biomarkers of diabetes and their inhibitors may be new targets for diabetes treatment.^[109]^ Additionally, PSTi8 (a pancreatin inhibitor) enhances mitochondrial function and the oxygen consumption rate in mouse WAT, thereby promoting energy expenditure.^[110]^ Another CgA-derived peptide, catestatin, is an insulin sensitizer^[111]^ and also promotes lipolysis.^[112]^

Elevated circulating levels of CgA are associated with nearly all types of neuroendocrine tumors, including PPGL. Serum CgA levels have an 83% to 89% sensitivity in diagnosing PCC,^[113]^ and up to 100% when combined with urinary catecholamines,.^[114]^ High levels of CgA may indicate a greater likelihood of malignancy in PPGL; moreover, CgA levels are also correlated with plasma metanephrine levels, which can be used to predict tumor responses to treatment and recurrence.^[115]^

However, no population-based studies have linked CgA or PST to energy expenditure, especially in PPGL-related populations. Furthermore, the overall biological effects of CgA may depend on its local concentration, protein hydrolysis process, and post-translational modifications, which may vary among tumors.^[96]^ Hence, caution is needed when defining the relationship between CgA and its derivatives, particularly PST, and high metabolic states observed in patients with PPGL.

## 5. Information suggested from hypermetabolism in PPGL

### 5.1. Need for careful diagnosis

Understanding the energy metabolism of patients with PPGL can help its diagnosis. The diagnostic significance of diabetes and weight loss in PPGL has long been recognized. In 2020, the European Society of Hypertension consensus proposed that PPGL should be strongly suspected in patients < 50 years of age with normal weight (BMI < 25 kg/m^2^) with both hypertension and diabetes. Biochemical monitoring is recommended in such patients.^[116]^

While PPGL diagnosis largely depends on imaging examinations, PET/CT has increasingly played a larger role in recent years, especially for patients with recurrence and metastasis. ^18^F-FDG-PET/CT is typically used to locate and monitor the invasive behavior of highly differentiated neuroendocrine tumors. However, activated BAT can sometimes be misinterpreted as a tumor on imaging, leading to false-positive results. Therefore, in FDG-PET/CT images, abnormalities in activated BAT areas should be carefully differentiated from those in other types of imaging images for accurate identification.

### 5.2. Hypermetabolic state is a potential indicator of PPGL prognosis

A high energy expenditure status could be closely related to the prognosis of patients with PPGL, especially a poor prognosis. Animals with higher energy expenditure or in a relatively hypermetabolic state tend to have shorter telomeres and lifespans.^[117]^ High metabolic states are common in patients with cancer, and increased REE is considered an early and major factor contributing to cachexia, which is often a poor prognostic indicator. A study evaluating the prognostic value of increased REE in a large cohort of patients with metastatic cell lung cancer found that hypermetabolism was associated with decreased survival; moreover, energy expenditure was an independent prognostic factor in a multivariate model for patients with cancer.^[118]^ Among 105 patients with hepatitis B virus-acute-on-chronic liver failure, Yao et al^[119]^ demonstrated that sustained hypermetabolism was an independent predictor of high short-term mortality. Patients with amyotrophic lateral sclerosis (ALS) show increased energy expenditure compared to a healthy control group according to metabolic indices, with hypermetabolic patients experiencing more severe functional decline and poorer survival rates.^[120]^ REE was also a valuable indicator for assessing the clinical outcomes of critically ill patients with sepsis in the intensive care unit with sepsis.^[121]^ Thus, metabolic indices may effectively predict disease outcomes.

However, research on the relationship between REE or other metabolic indicators and survival rates in PPGL is lacking. BAT activation is associated with decreased overall survival in PPGL, with the mortality rate for BAT activation approximately 5.8 times that of the control group. The present study observed a significant correlation between BAT activating with higher plasma norepinephrine levels and low mortality rates. Because the norepinephrine levels of deceased patients in the BAT group were all above the 75th percentile of the norepinephrine range, and due to limitations in sample size, subgroup analysis could not be performed to determine the individual impact of BAT on mortality. The adverse outcomes observed in patients with BAT activation were likely due to the weakness mediated by energy expenditure.^[70]^ Therefore, further research is needed to clarify the separate effects and the precise mechanisms of BAT activation on the survival of patients with PPGL and to determine whether BAT activation and REE increase can serve as prognostic factors for patients with PPGL.

Untreated patients with PPGL and excessive catecholamine levels can result in the activation of their respective receptors, leading to a series of effects. Chronic stress can promote tumor growth through adrenergic receptors. A previous review summarized the role of chronic stress in the occurrence and development of tumors and suggested that excess stress hormones promote cancer by inducing the accumulation of DNA damage, increasing P53 degradation, and other related pathways. These hormones can also enhance inflammation, suppress immunity, prevent immune cells from effectively controlling cancer cells, and influence stromal cells in the tumor microenvironment, thereby promoting tumor growth, invasion, and metastasis.^[122]^

Also noteworthy is the ongoing surge of research on brown fat activation and white fat browning, particularly in the context of metabolic regulation and obesity resistance. Therapeutically activating BAT to increase energy expenditure by stimulating fat utilization has gained attention.^[123]^ Thus, PPGL may serve as a natural human model for inducing BAT activation.

## 6. Coping with hypermetabolism with PPGL

Most studies have indicated that patients may experience postoperative weight gain; hence, surgical removal of lesions remains the primary approach to address the chronic hypermetabolic state in patients with PPGL. Case reports suggest that α-adrenergic receptor blockers may be effective.^[79]^ Research on another hypermetabolic state, burns, showed that β-adrenergic receptor blockers can reduce REE and improve energy metabolism.^[124–126]^ In patients with PPGL, the use of β-blockers depends on the tachycardia induced by catecholamines or reflex tachycardia caused by phenylephrine.^[127]^ Therefore, their use is based on symptom severity and medical preferences and no exact ratio data is currently available. Adequate nutrient intake may help counter disease-related consumption and promote postoperative recovery.

## 7. Limitations

The limitations of this review primarily arise from the relatively small number of studies on carbohydrate, lipid, protein and energy metabolism in patients with PPGL. As the overall incidence of PPGL is relatively low, large-scale studies are challenging. Moreover, overweight or obesity, medication and diet habits, as well as other chronic metabolic disease, can affect metabolism status. Additionally, all factors may occur simultaneously, leading to metabolic overactivity, making it difficult to quantify the isolated impact of single factor on energy expenditure.

## 8. Conclusions

Chronic hypermetabolism can lead to severe organ, muscle, protein, and fat loss, along with hepatic steatosis and immune suppression,^[33]^ and can even patient life expectancy. While hypermetabolism in patients with PPGL is not rare, it has received insufficient attention. While the effects of catecholamines on energy and substance metabolism have been extensively studied under physiological conditions, this scenario changes under pathological conditions. Although patients with PPGL show BAT activation and inflammatory states, quantified results linking these findings to energy expenditure are lacking. Furthermore, unlike general tumors, endocrine-related tumors generally have a relatively favorable prognosis. For PPGL, the 5-year survival rate after surgery for non-metastatic tumors can reach 95%, whereas, for tumors that have metastasized, the 5-year survival rate is approximately 30–40%^[128]^; thus, factors contributing to cachexia require confirmation.

We categorized the possible mechanisms of energy hypermetabolism in PPGL into five categories: catecholamines, BAT activation, inflammation, CgA, and its derivatives, and their impact on tumor behavior. However, in real-world settings, these aspects are not parallel but rather interact within the body. Further clinical research on PPGL-related energy aspects, especially large-scale, multicenter prospective cohort studies, is needed to guide more precise PPGL diagnosis and treatment.

## Acknowledgments

The authors are grateful to the patients for their kind contribution to this study.

## Author contributions

**Conceptualization:** Yuqi Yang, Tong Zhou, Guixia Wang.

**Funding acquisition:** Tong Zhou, Guixia Wang.

**Investigation:** Yuqi Yang, Xue Zhao, Yao Xu.

**Methodology:** Yuqi Yang, Tong Zhou, Xue Zhao, Yunjia Cai, Xiaokun Gang, Guixia Wang.

**Validation:** Xiaokun Gang, Guixia Wang.

**Visualization:** Yuqi Yang, Yunjia Cai, Yao Xu.

**Writing – original draft:** Yuqi Yang.

**Writing – review & editing:** Tong Zhou, Xue Zhao, Guixia Wang.
